# The differential presence of human polyomaviruses, JCPyV and BKPyV, in prostate cancer and benign prostate hypertrophy tissues

**DOI:** 10.1186/s12885-021-08862-w

**Published:** 2021-10-24

**Authors:** Chenghuang Shen, Chunliang Tung, Chunnun Chao, Yeongchin Jou, Shupei Huang, Menghsiao Meng, Deching Chang, Peilain Chen

**Affiliations:** 1 Department of Urology, Chiayi Christian Hospital, Chiayi, Taiwan; 2 Department of Pathology, Chiayi Christian Hospital, Chiayi, Taiwan; 3 Department of Health and Nutrition Biotechnology, Asian University, Taichung, Taiwan; 4 Department of Pediatrics, Chiayi Christian Hospital, Chiayi, Taiwan; 5 Department of Medical Laboratory Science and Biotechonology, Asian University, Taichung, Taiwan; 6 Department of Medical Laboratory Science and Biotechnology, Central Taiwan University of Science and Technology, Taichung, Taiwan; 7 Graduate Institute of Biotechnology, National Chung Hsiang University, Taichung, Taiwan; 8 Institute of Molecular Biology, National Chung Cheng University, Chiayi, Taiwan

**Keywords:** Prostate cancer (PC), Benign prostate hypertrophy (BPH), JCPyV/BKPyV, cancer progression and prognosis

## Abstract

**Background:**

Studies have shown that human polyomavirus infection may be associated with various human cancers. We investigated the potential relationship between the prevalence of JCPyVor BKPyV and prostate cancer (PC) in patients from Taiwan.

**Methods:**

Patients with PC and benign prostate hypertrophy (BPH; 76 and 30 patients, respectively) were recruited for this study. Paraffin-embedded tissues and clinical information of the patients were obtained. The tissue sections were used for viral DNA detection and immunohistochemistry analysis was performed for examining viral large T (LT) and VP1 proteins. Regression analysis was used to evaluate the relationship between the clinical characteristics of the patients and the risk of JCPyV/BKPyV infection.

**Results:**

The prevalence of JCPyV/BKPyV DNA was different in PC and BPH tissues (27/76 [35.52%] and 2/30 [6.7%], respectively, *p* = 0.003)]. The LT and VP1 proteins were detected in 27 (35.52%) and 29 PC (38.2%) specimens, respectively, but neither protein was detected in BPH samples (*p* < 0.001). PC cells were more susceptible to JCPyV infection than BPH tissues [odds ratio (OR) 7.71, 95% CI: 1.71–34.09, *p* = 0.003). Patients with PC showing high levels of prostate-specific antigen and high Gleason scores were associated with a high risk of viral infection (ORs 1.1, 95% CI 1.000–1.003; *p* = 0.045 and ORs 6.18, 95% CI 1.26–30.33, *p* = 0.025, respectively). The expression of LT protein associated with the risk of PC increased 2923.39-fold (95% CI 51.19–166,963.62, *p* < 0.001).

**Conclusions:**

The findings indicate that JCPyV infection in PC cells may be associated with prostate cancer progression and prognosis.

**Graphical abstract:**

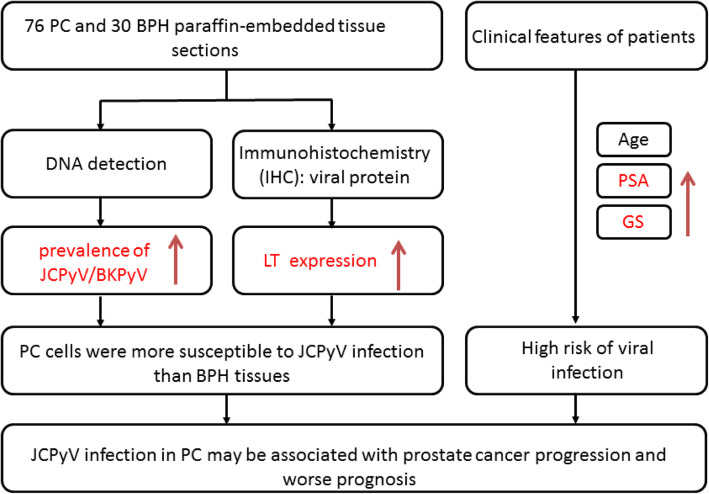

**Supplementary Information:**

The online version contains supplementary material available at 10.1186/s12885-021-08862-w.

## Background

Prostate cancer (PC) has the second-highest incidence and results in the fifth-highest mortality rates among men worldwide [1]. In Taiwan, the mortality resulting due to PC ranks seventh among all cancers, and the incidence ranks fourth, with 6644 of 100,000 men diagnosed with PC annually [2], making it a health concern that is drawing increasing attention. Early symptoms of PC are similar to those of benign prostate hypertrophy (BPH). In early PC, the prostate hypertrophies and compresses the urethra, resulting in difficulty with urination, oliguria, polyuria, and incomplete bladder emptying; these symptoms are not serious. In later stages of PC development, hematuria, pelvic pain, urinary incontinence, and other symptoms may develop [3]. Currently, biomarkers are utilized for clinical diagnosis of patients with PC, which include blood-based prostate-specific antigen (PSA) [4], Prostate Health Index (PHI) [5], 4 K score (total PSA, free PSA, intact PSA, and human kallikrein-related peptidase 2) [6]; urine-based- PCA3(prostate cancer gene 3) [7], SelectMDx(detection of HOXC6 and DLX1 mRNA levels) [8], ExoDx Prostate IntelliScore (liquid biopsy test indicated for men 50 years of age and older with a PSA 2–10 ng/mL, or PSA in the “gray zone”, considering an initial biopsy) [9, 10]. In addition, commercial gene expression assays guide clinical prognosis and treatment in patients with PC, particularly when considering active surveillance. Prolaris test (detection of 31 regulatory genes of cell cycle and 15 housekeeping genes) for predicting the recurrence of the disease and survival of patients is employed after radical prostatectomy [4, 11]. The Oncotype DX test (detection of 12 tumor-associated genes and 5 housekeeping genes) assesses high-risk patients with higher Gleason scores after prostate treatment [4, 12]. Decipher test (detection of 22 genes, including those corresponding to cell proliferation, differentiation, immune regulation, androgen signaling pathways) can be used as an independent prognostic factor of postoperative metastasis [4, 13]. AR-V7 (androgen receptor variant 7 protein) is a treatment-specific biomarker for patients with metastatic castrate-resistant PC [4, 14, 15]. However, PSA is the most commonly used serological marker for clinical screening of patients with prostate cancer and prostate inflammation or hypertrophy. The Gleason score is an important indicator for evaluating the prognosis of PC, with higher scores indicating a worse prognosis [3]. The risk factors for PC include age, race, the prevalence of genetic mutations, hereditary factors, chronic inflammation, and infection [1, 16]. A few studies have examined pathogens that mediate chronic inflammation of the prostate and accelerate PC development [17–19]. Viruses with tissue tropism that have been latent or proliferating in prostate tissue and the genitourinary tract for a long time have been implicated in the increase observed in the incidence of PC [20–23].

JC polyomavirus (JCPyV) and BK polyomavirus (BKPyV) are two common human polyomaviruses. They are non-enveloped DNA viruses with similar genomes and DNA sequences [21], and their genomes contain both early and late transcriptional regions. The early region encodes the oncogenic proteins large T (LT) antigen and small t antigen, whereas the late region encodes the structural proteins VP1, VP2, and VP3, and agnoprotein. The regulatory regions of JCPyV and BKPyV contain the viral DNA origin of replication, promoter, enhancer, and cell transcription factor binding sites. Different genotypes of the same viral strain form new rearranged variants via mutations in regulatory regions that may include point mutations, deletions, duplications, and alterations [24]. These mutations enable the virus to adapt to changes in the host environment, increase or decrease the affinity of viral DNA to cellular transcription factors, or act as a cofactor for carcinogenesis in tissue-tropic cells [24]. When a polyomavirus infects permissive cells, it produces and assembles viral progeny in the nucleus. Subsequently, the host cells are lysed, resulting in fatal progressive multifocal leukoencephalopathy or fatal nephritis and hemorrhagic cystitis in cases with JCPyV [21, 25] or BKPyV infection [21, 26], respectively. The LT protein can interact with the tumor suppressor proteins p53 and Rb, resulting in uncontrolled cell proliferation [27]. Recent studies have indicated that both the JCPyV and BKPyV genomes are found in human tumors [21]. JCPyV has been associated with brain cancer, colorectal cancer, lymphoma, central nervous system tumors, esophageal cancer, bladder cancer, and PC, whereas BKPyV has been associated with pancreatic cancer, Kaposi’s sarcoma, bladder cancer, rhabdomyosarcoma, PC, and head and neck tumors.

No relevant studies examining JCPyV and BKPyV infections in association with PC and BPH among Taiwanese patients have been reported. The purpose of this study was to investigate the possible associations between JCPyV and BKPyV viral infections and PC in patients from Taiwan. We aimed to evaluate (1) the rates of JCPyV and BKPyV infections in PC and BPH tissues in patients from Taiwan between 2016 to 2020, and (2) the correlation between the clinical characteristics of patients and the risk of JCPyV/BKPyV infection in PC cells.

## Methods

### Subjects

We recruited patients with PC and BPH using intentional sampling at the Department of Urology, Chiayi Christian Hospital in Taiwan from 2016 to 2020. The inclusion criteria were as follows: (1) patients diagnosed with PC or BPH; (2) tissue samples with cancerous area higher than 50%; (3) completion of the surveys by patients with or without help. The exclusion criteria were as follows: (1) patients with a history of other cancers; (2) severe communication problems; (3) patients with missing clinically important pathological data; and (4) patients who were immunocompromised or had post-transplanted organs. All patients provided written informed consent, and their confidentiality was safeguarded. Specimens were collected after review, and the study was approved by the institutional review board of Chiayi Christian Hospital.

### Data collection

The clinical data of the patients in this study were used to investigate the relationship between JCPyV and BKPyV with PC and BPH in Taiwan. The patient clinical-pathological information of the patients including age, sex, tumor size, tumor tissue types, tumor-node-metastasis (TNM) stage, prostate-specific antigen (PSA) concentration, Gleason score of the patients with PC, and the age, sex, and PSA concentration of patients with BPH were collected. All patients with PC were characterized with clinical-pathological stages between Tx and T4, as defined in the American Joint Committee on Cancer (AJCC) TNM staging system for PC [28]. A total of 76 prostate adenocarcinoma tissues and 30 BPH tissue specimens were collected, thoroughly cleaned, and processed independently for analysis. PC specimens were obtained using transrectal ultrasound biopsy, transurethral biopsy, and radical prostatectomy. BPH specimens were obtained via transurethral resection of the prostate for bladder outlet obstruction. The specimens were stored in the pathology department until analysis. Pathological tissue sections were used to investigate the presence of the JCPyV and BKPyV genomes and the expression of the viral proteins LT and VP1. A formalin-fixed, paraffin-embedded JCPyV-bearing cell line (JCI) [29, 30] was used as a positive control for PCR and immunohistochemical staining.

### DNA extraction

DNA samples were extracted from paraffin-embedded tissue using the Gene JET FFPE DNA purification reagent (Thermo Fisher, Vilnius, Lithuania) according to the instructions of the manufacturer. Briefly, tissue was deparaffinized using xylene, washed 2–3 times with absolute ethanol, washed with deionized water to remove residual ethanol, and air-dried. The sample was treated with 50 μg/ml proteinase K (Thermo Fisher, Vilnius, Lithuania) for 16–18 h at 50 °C, placed in boiling water for 10 min to deactivate proteinase K, and centrifuged at 11,180×*g* for 3 min. The supernatant was collected for DNA purification. DNA (200 ng) was collected for viral genome analysis using PCR [29]. The human β-actin gene was used as an internal control to ensure successful DNA extraction from paraffin-embedded tissues and to exclude false negatives.

### Nested PCR and DNA sequencing

Nested PCR was used to detect the presence of viral DNA in PC and BPH specimens. Typically, after polyomavirus infection, the genome may undergo rearrangement within regulatory regions to produce new variants [31]. Therefore, we used two primer pairs to amplify the constant regulatory regions of JCPyV and BKPyV [29]. The first PCR used the primer pair JBR1 and JBR2 (nucleotides 5067–5091 of the JCPyV TW-3 strain, 5′-CCTCCACGCCCTTACTACTTCTGAG-3′ and 279–255, 5′-GTGACAGCTGGCGAAGAACCATGGC-3′, respectively) to amplify the regulatory region. Next, 5 μL of the first PCR product was used as the template, and the primer pair JBRNS and JBRNAS (nucleotides 5100–5 of the JCPyV TW-3 strain, 5′-GAGGCGGCCTCGGCCTC-3′ and 227–212, 5′-GGCTCGCAAAACATGT-3′, respectively) were used for the second PCR. PCR amplification of the human beta-globin (β-globin) 5′ UTR region was performed simultaneously to ensure the presence of tissue DNA and exclude false negative results. All specimens were analyzed in triplicate. The secondary PCR products were analyzed via electrophoresis using 2.5% agarose gels. The JCPyV CY (GenBank Accession No. AB03849), and BKPyV UT (GenBank accession No. M 34049.1) genotypes were used as positive controls. The nested PCR products had a size of 243 bp and 289 bp, respectively, which were consistent with those of the regulatory regions of JCPyV and BKPyV. The PCR products were sent to Mingxin Biotechnology (Taipei City, Taiwan) for DNA sequencing analysis after purification. All sequences were compared with the JCPyV archetype (GenBank accession No. AB03849), and BKPyV prototype (GenBank accession No. M 34049.1) to confirm whether the regulatory regions underwent rearrangement and to determine the genotypes.

### Immunohistochemistry (IHC)

IHC staining was performed as previously reported [29] with minor modifications. Briefly, 3 μm thick formalin-fixed, paraffin-embedded tissue sections were deparaffinized with xylene for 10 min, rehydrated through a gradient of 100, 95, 80, and 70% ethanol, and equilibrated in Tris buffer (TBS; 0.1 M Tris-HCl, pH 7.4 and 0.15 M NaCl). Antigen retrieval was performed by placing the slides in 0.01 M citric buffer (pH 6.0) and autoclaving at 121 °C for 20 min. The slides were treated with a protein-blocking agent for 10 min to reduce the non-specific binding of antibodies to the tissue and subsequently washed twice. Primary monoclonal antibodies anti-SV40 LT (mia90661; Thermo Fisher, Vilnius, Lithuania) and anti-JCPyV capsid VP1 (ab34756; Abcam, Cambridge, USA) were used to detect the expression of the early viral protein LT and the late structural protein VP1, respectively. Tissue sections were incubated overnight in a humidified chamber at 37 °C, followed by incubation after the sequential addition of biotinylated secondary antibody (PK6102, Vectastain ABC kit, Burlingame, CA, USA) and avidin-biotin complex (Vectastain ABC kit, Burlingame, CA, USA) for 1 h each. After development in the presence of diaminobenzidine (DAB) substrate (Sigma–Aldrich, St. Louis, MO, USA), sections were counterstained using hematoxylin.

### Transmission electron microscopy (TEM)

Four fresh tissue samples were collected from 27 PC tissues that were positive for JCPyV DNA and VP1 protein expression. The specimens that were fixed in 2.5% glutaraldehyde were cut into cubes of approximately 1 mm^3^. These specimens were dehydrated and embedded in LR White Resin (Polysciences, Warrington, PA, USA). The embedded specimen was subsequently sliced into 60 nm-thick sections and attached to nickel grids. After incubation in 0.25% gelatin-containing TBST buffer for 1 h, the grids were sequentially incubated with anti-JCPyV VP1 monoclonal antibody (ab34756; Abcam, Cambridge, USA) for 2 h and with 15 nm gold-conjugated protein-G (EM.PGG15; Agar, Essex, UK) for 1 h. The immunolabeled sections were treated with 2% uranyl acetate for 20 min and 0.5% lead citrate for 10 min and observed using a JEM-100CX II transmission electron microscope (JEOL, Peabody, MA, USA) [32].

### Statistical analysis

All statistical analyses were conducted using SPSS version 18.0, for Windows (SPSS Inc., Chicago, IL, USA). Statistical significance was set at *p* < 0.05. Fisher’s exact test or the Mann-Whitney U test was used to analyze the differences between the prevalence of viral DNA and LT and VP1 proteins in PC and BPH tissues. Continuous evaluations were compared between groups using the Mann-Whitney U test. Fisher’s exact test was used to compare categorical variables. The relationship between the clinical characteristics of patients and the risk of JCPyV/BKPyV infection in PC cells, the odds ratio, and 95% confidence interval were evaluated using logistic regression analysis.

## Results

### Clinical characteristics of patients with PC and BPH

A total of 106 men (76 and 30 patients with PC and BPH, respectively) were included in the study. The clinical characteristics of the patients with PC, including age, TMN staging, PSA, and Gleason score, are described in Additional file 1: Table S1, and similar information for patients with BPH is included in Additional file 2: Table S2. We analyzed the differences in clinical-pathological characteristics between patients with PC and those with BPH, and the results are shown in Table 1. The average age of patients with PC or BPH was 73.4 ± 9.2 years or 70.9 ± 6.0 years, respectively, and the difference was not significant (*p* = 0.258). The Gleason score for pathological diagnosis was > 6 in all patients with PC. There were 3 (4.0%), 19 (25.0%), 26 (34.2%), and 28 (36.8%) patients with scores of 10, 9, 8, and 7, respectively. The average PSA values in the PC and BPH groups were 584.4 ± 2055.9 and 6.9 ± 4.5 ng/mL, respectively, and the difference was significant (*p* < 0.001).

**Table 1:** The pathological features associated with PC and BPH among 106 study subjects

Variables	PC	BPH	***p***-value
	*N* = 76	*N* = 30	
Age (years)	73.4 ± 9.2	70.9 ± 6.0	0.258
PSA (ng/mL)	584.4 ± 2055.9	6.9 ± 4.5	< 0.001*
GS			
10	3 (4.0%)	x	
9	19 (25%)	x	
8	26 (34.2%)	x	
7	28 (36.8%)	x	

The *p*-value was determined using the Mann-Whitney U test. Asterisks indicate *p-*values with statistical significance. For the age and PSA analysis, continuous variables are shown as mean ± SD; for GS (Gleason score) in PC: scores 10–7 are presented as numbers and percentages

### Presence of viral DNA in PC and BPH tissue samples

**Fig. 1:**
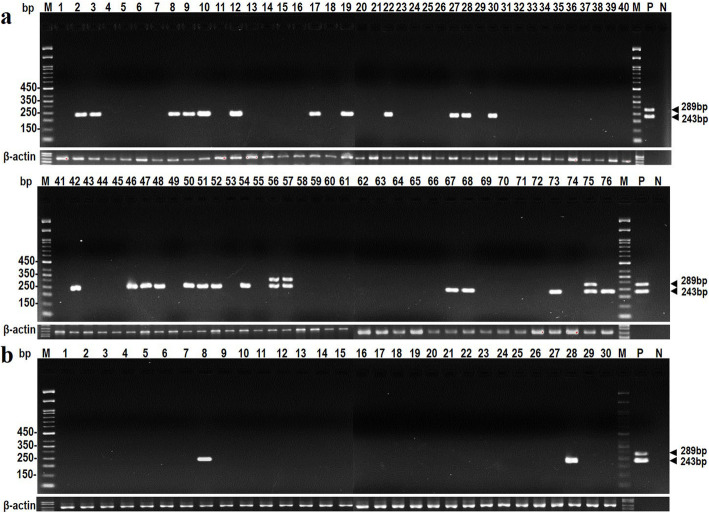
Detection of JCPyV and BKPyV viral DNA in prostate cancer and benign prostate hypertrophy specimens. The viral regulatory region was detected by nested PCR using the conserved JCPyV and BKPyV primers. The PCR products were analyzed on a 2.5% agarose gel with molecular markers. Lane M: 50 bp DNA molecular ladder. Lane numbers represent the number of tissue samples from prostate cancer (**a**) and BHP tissues (**b**). Lane P: The PCR products amplified from the JCPyV CY and BKPyV genomic DNA were used as positive controls and are indicated by arrows at 243 bp and 289 bp, respectively. PCR with no DNA template was used as a negative control. The PCR product of β-actin was used as an internal control

Nested PCR was used to detect the presence of JCPyV and BKPyV regulatory regions in all 106 PC and BPH tissue specimens. The results are shown in Fig. 1, and Additional file 3: Fig. S1. In 24 of the 76 PC specimens, a DNA product with a length of 243 bp was amplified, which is consistent with the size of the DNA amplified from JCPyV DNA. In 3 specimens, DNA products of sizes 243 bp and 289 bp were amplified, which is consistent with the prevalence of JCPyV and BKPyV (Fig. 1a). PCR results showed that 2 of the 30 BPH specimens alone were positive for infection (Fig. 1b). All PCR products were confirmed by sequencing, and 24 of the 27 PC-positive specimens contained JCPyV, and three contained both JCPyV and BKPyV, with a positive rate of 35.5% (27/76) (Table 2). The two positive BPH samples contained JCPyV resulting in a positive rate of 6.7% (2/30) (Table 2). Fisher’s exact test analysis indicated that the presence of JCPyV and BKPyV viral DNA in PC tissues was higher than that in BPH tissues (*p* = 0.003) (Table 2).

**Table 2:** The prevalence of viral DNA and proteins in PC and BPH tissues

Viral targets	PC ***N*** = 76 (100%)	BPH ***N*** = 30 (100%)	***p-***value
DNA	27 (35.5%)	2 (6.7%)	0.003 *
LT	27 (35.5%)	0 (0%)	< 0.001*
VP1	29 (38.2%)	0 (0%)	< 0.001*

The *p*-value was determined using Fisher’s exact test. Asterisks indicate *p-*values with statistical significance, and categorical variables are reported as numbers and percentages

### Genotyping of viral DNA in PC and BPH tissue samples

Viral DNA identified using PCR in PC and BPH tissues were compared with the archetypal JCPyV-CY (GenBank accession No. AB03849), and the prototypal BKPyV-UT strains (GenBank accession No. M34049.1). The results are shown in Additional file 4: Fig. S2, which indicates that most specimens contained deletion mutations in nucleotides 195–199 and 224–228 within the regulatory region, indicating similarity to the JCPyV TW-3 strain (GenBank accession No. U61771). Among the PC specimens, 21 were JCPyV TW3 strain-like, 1 each were JCPyV CY strain-like and JCPyV SK3 strain-like (GenBank accession No. AB118652.1), and three specimens showed two genotypes, JCPyV TW3 strain-like and BKPyV UT strain-like (Fig. S2. a-d). Among the two positive BPH specimens, one was JCPyV CY strain-like, and the other was JCPyV TW3 strain-like (Fig. S2. e). Genotyping results are shown in Table 3; JCPyV was the major virus strain in PC-positive samples at 88.9% (24/27), of which 3.7% were CY-like (1/24), 81.5% were TW3-like (22/27), and 3.7% were SK3-like (1/24). The genotypes containing both JCPyV and BKPyV were TW3-like and UT-like, accounting for 11.1% (3/27) of positive samples. There were two positive JCPyV cases in the BPH tissues, namely TW3-like and CY-like, each accounting for 50% (1/2) of the positive BPH samples. Compared to the original sequences, these genotypes had different nucleotide mutations.

**Table 3:** Distribution of the genotypes for JCPyV and BKPyV in PC and BPH samples

Genotypes	PC	BPH
	***N*** = 27 (100%)	***N*** = 2 (100%)
**JCPyV**	**24 (88.9%)**	**2 (100%)**
TW3-like	22 (81.5%)	1 (50%)
CY-like	1 (3.7%)	1 (50%)
SK3-like	1 (3.7%)	0 (0%)
**JCPyV + BKPyV**	**3 (11.1%)**	**0 (0%)**
TW3-like + UT-like	3 (11.1%)	0 (0%)

### Detection of viral proteins in PC and BPH tissue samples

**Fig. 2:**
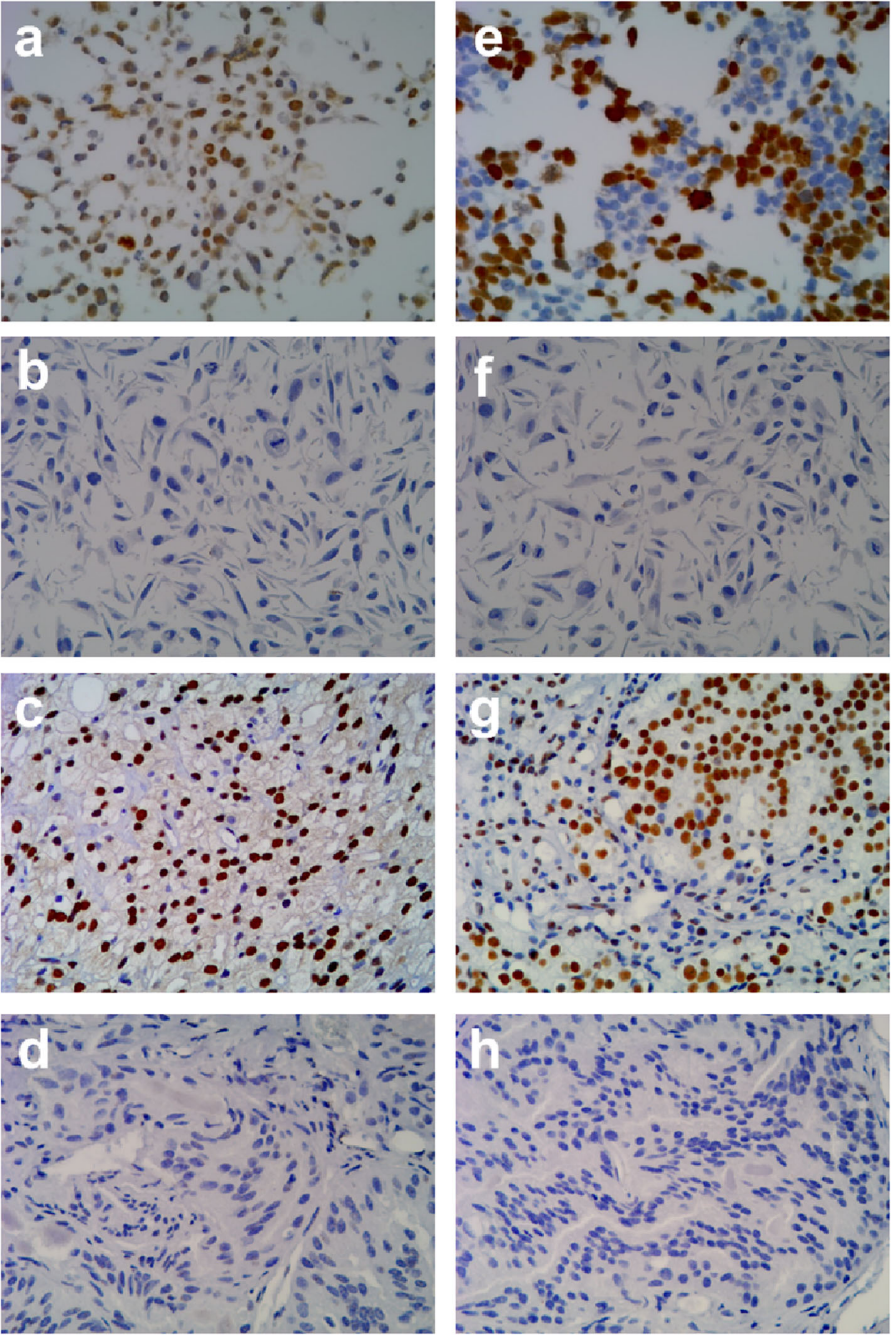
Detection of viral proteins in the prostate tissues by immunohistochemical staining. Sections of prostate cancer (PC) tissues were stained with anti-SV 40 LT monoclonal antibody (**a**-**d**) or anti-JCVP1 monoclonal antibody (**e**-**h**). JCI cells from the JCPyV carrier cell line were used as a positive control (panels **a** and **e**). IMR 32 cells were used as a negative control (panels **b** and **f**). Panels c and g show LT and VP1-positive staining, respectively, in the section for PC tissue number 76. Panels d and h show LT and VP1-negative staining, respectively, in the section for PC tissue number 39. Original magnification × 200

Figure 2 shows the IHC staining results for detecting the expression of early viral protein LT and the late structural protein VP1 in PC and BPH tissues. The LT and VP1 proteins were detected in the nuclei of cancer cells and were absent in neighboring normal cells. Of the 76 PC specimens, 27 were positive for LT (Additional file 1: Table S1), with a positive rate of 35.5% (Table 2), whereas no LT protein expression was detected in BPH tissues (Additional file 2: Table S2), and the difference was significant (*p* < 0.001) (Table 2). In addition, 29 PC tissue specimens were positive for VP1 protein (Additional file 1: Table S1), with a positive rate of 38.2% (29/76) (Table 2), whereas VP1 protein expression was not detected in the BPH samples (Fig. 2, Additional file 2: Table S2) (*p* < 0.001) (Table 2).

**Fig. 3:**
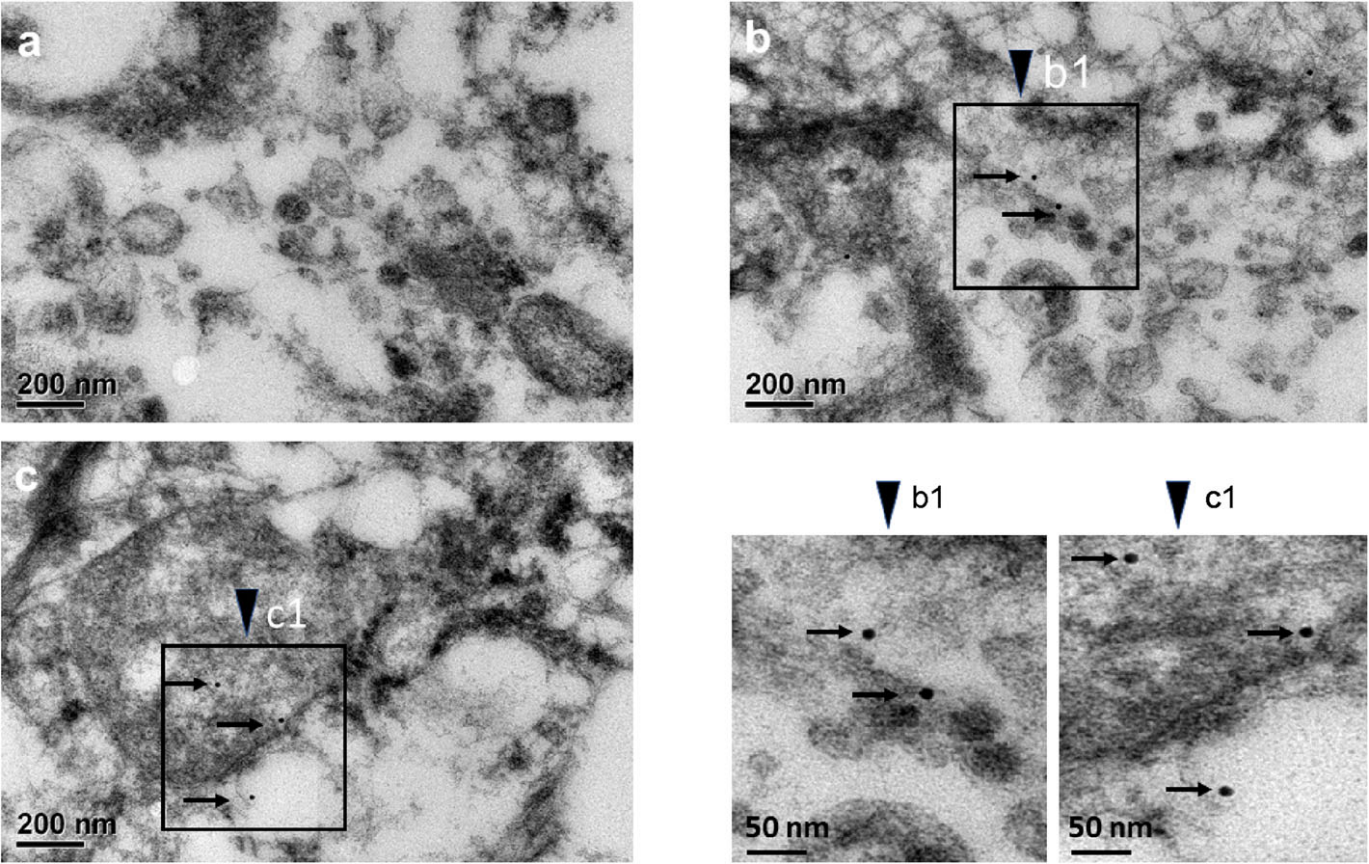
Detection of VP1 of JCPyV in prostate tissues by electron microscopy analysis. Tissue sections were treated using a standard immunogold-labeling protocol [32]. The presence of JCPyV in the tissue is indicated by the 15 nm gold particles. (**a**) The use of negative technical control was omitted for the incubation step with anti-JCPyV VP1 monoclonal antibody in the labeling protocol. No gold particles were detected in the control sample. (**b**, **c**) Tissue sections were treated using the standard labeling protocol. The thin arrowheads point to gold particles that indirectly indicate the presence of the viral VP1 protein

To confirm the presence of JCPyV capsid proteins in PC tissues, TEM was used to observe their expression. Anti-JCPyV VP1 monoclonal antibody was used for immunogold labeling. Gold particles (15 nm) were observed in the prostate cancer tissues (Fig. 3b, b1, c, c1), whereas a negative result was observed in similarly treated PC tissues without exposure to the primary JCPyV monoclonal antibody (Fig. 3a). The results indicate that the viral capsid VP1 protein is present in PC tissues.

### Comparison of the risk of JCPyV and BKPyV infection in PC and BPH

Based on the results mentioned above for the detection of viral DNA and protein in PC and BPH tissues, we used Fisher’s exact test to compare the differences between JCPyV and BKPyV infection. The results are presented in Table 4 and showed that 27 of 76 PC tissues (35.5%), and 2 of 30 BPH tissues had JCPyV infection (6.7%). BKPyV-infected PC accounted for 3.9%, and no BKPyV-infected BPH. Binary logistic regression analysis indicated that the risk of JCPyV infection in PC was higher than for BPH by 7.71-fold (95% CI: 1.71–34.09, *p* = 0.003).

**Table 4:** Comparison of the risk of JCPyV and BKPyV infection in PC and BPH tissues

Variables	PC	BPH	***p-***value	ORs (95%CI)
	***N*** = 76	***N*** = 30		
JCPyV(+)	27 (35.5%)	2 (6.7%)	0.003*	7.71 (1.71–34.09)
BKPyV(+)	3 (3.9%)	0 (0.0%)	0.557	–

The *p*-value was determined using Fisher’s exact test. Asterisks indicate *p-*values with statistical significance, and categorical variables are reported as numbers and percentages. Odds ratios (ORs) and 95% confidence intervals (CIs) were calculated using logistic regression analysis

### The correlation between clinical characteristics of patients with PC and risk of JCPyV/BKPyV infection

Binary logistic regression analysis was used to analyze the relationship between clinical-pathological characteristics of the 76 patients with PC (including 27 cases with and 49 cases without viral infection), including PSA biomarker concentration, Gleason score, and the risk of JCPyV infection. The results of the omnibus test of model coefficients were χ^2^ = 70.752***, *p* < 0.001, as shown in Table 5. The PSA concentration was significantly associated with the risk of JCPyV infection in PC. The risk of infection increased 1.1-fold for every unit increase in PSA concentration, compared to patients without viral infection (95% CI 1.000–1.003; *p* = 0.045). In patients with PC with Gleason scores of 7–10, statistical analysis indicated that a high Gleason score was associated with a significantly increased 6.18-fold risk for JCPyV infection (95% CI 1.26–30.33, *p* = 0.025) compared to patients without viral infection. In addition, when the LT protein was present, the risk of PC was increased by 2923.39-fold (95% CI 51.19–166,963.62, *p* < 0.001).

**Table 5:** Binary logistic regression analysis of association between JCPyV/BKPyV infection risk and PC patients’ clinical features

Variables	β	ORs	(95% CI)	***p***-value
PSA	0.001	1.10	(1.000–1.003)	0.045*
GS	1.821	6.18	(1.26–30.33)	0.025*
LT	7.980	2923.39	(51.19–166,963.62)	< 0.001***

Omnibus test: χ^2^ = 70.752, *** *p* < 0.001; Odds ratios (ORs) and 95% CIs were used as variables for PSA, GS, and LT for binary logistic regression analysis. Asterisks indicate *p-*values with statistical significance, * *p* < .05, ** *p* < .01, *** *p* < .001

## Discussion

In this study, we evaluated the rate of infection by human polyomaviruses JCPyV and BKPyV in PC cells in patients from Taiwan evaluated between 2016 and 2020. We found that 35.2% (27/76) of the PC specimens were positive for viral DNA. JCPyV infection was present in 88.9% of samples (24/27), whereas simultaneous JCPyV and BKPyV infections were present in 11.1% (3/76) of PC tissues. However, only 6.7% (2/30) of the BPH specimens were positive for viral DNA, all of which were infected with JCPyV, and BKPyV DNA was not detected. The most common strain among the virus-infected PC samples was JCPyV TW3, accounting for 85.2% (23/27) prevalence. Our results indicate that JCPyV and BKPyV DNA and protein are distributed differently within PC and BPH tissues and that PC cells are more susceptible to JCPyV/BKPyV infection than benign tissues. In addition, we assessed the relationship between the risk and clinical characteristics of patients with PC and BPH. No difference in age was observed between the patients infected with either of the two diseases; however, the average PSA concentration and the risk of JCPyV infection were significantly higher in patients with PC than with BPH. Furthermore, PSA and Gleason scores were correlated with the risk of JCPyV infection in PC cells, and this risk was higher when the LT protein was present. Viral infection of PC cells may enhance PC progression, which is in turn related to the patient’s prognosis. The incidence of PC varies according to geographical distribution [1]. In Europe and Africa, where the incidence and mortality of PC are high, BKPyV is the major polyomavirus infection associated with PC. In Asia, where PC incidence and mortality are increasing each year [1], our results evaluating patients from Taiwan indicated that JCPyV infection is significantly more common than BKPyV infection. In addition, the rates of JCPyV infections in other tumor tissues, such as DLBCL [29], bladder cancer [33], and rectal cancer [34] tissues in patients from Taiwan, are higher than those of BKPyV infections. Therefore, polyomaviruses may infect cancer cells with geographical and regional specificity.

IHC analysis showed that 35.2% (27/76) of the specimens were positive for the LT viral protein and 38.2% (29/76) were positive for VP1. TEM analysis identified four VP1-positive cases, and colloidal gold-labeled viral capsid proteins were detected in fresh samples in the presence of JCPyV DNA. These results indicate that JCPyV infects PC cells via a lytic infection pathway. This is the first report depicting infection of PC cells by polyomavirus JCPyV in contrast to previous findings. Previously, infection of PC cells by polyomaviruses was primarily demonstrated via latent infection. A few studies have shown that LT has a p53 binding site, enabling it to bind p53 in the cell and inhibit its function, resulting in carcinogenesis [21, 35, 36]. In this study, to ensure the accuracy of viral DNA and protein analysis, we collected tissue samples in which the cancerous areas were higher than 50%. The Gleason score for pathological diagnosis was > 6 for all patients with PC. Logistic regression analysis was used to analyze the association between patients with high grade (GS 7–10 score) PC and the risk of JCPyV infection. Our results showed that the risk of PC infection increased by 6.18 fold for every 1 unit score increase in GS and with the expression of LT protein. Previous studies examining mRNA expression to predict the progression of patients with PC showed that patients with high-grade PC (GS > 7) demonstrated high mRNA expression of specific genes [4, 7, 12, 37]. We suspected that when JCPyV infects PC cells, its oncoprotein LT may enhance cellular mRNA expression of specific genes and may affect GS score and progression of PC. However, whether the changes after viral infection are associated with the opinion remains to be investigated.

## Conclusions

We analyzed JCPyV and BKPyV infections in 76 and 30 patients with PC and BPH, respectively, in Taiwan. Our results showed that JCPyV and BKPyV DNA and protein are distributed differently within PC and BPH tissues and that PC cells are more susceptible to JCPyV/BKPyV infection than benign tissues. JCPyV infects PC cells via a lytic infection pathway. Subjects with high PSA concentrations and high Gleason scores were significantly associated with the risk of JCPyV infection. In addition, the presence of LT protein increases the risk of PC. Our findings may provide evidence for the association of JCPyV infection in PC cells with prostate cancer progression and prognosis. More research is needed to understand the mechanisms underlying risk factors for PC and treatments that may improve the outcomes for PC in the future.

## Supplementary Information

**Additional file 1: Table S1.** Characteristics of prostate cancer (PC) samples and summary of the analysis of human polyomavirus (JCPyV and BKPyV) DNA and proteins.

**Additional file 2: Table S2.** Characteristics of benign prostate hypertrophy (BPH) samples and summary of the analysis of human polyomavirus (JCPyV and BKPyV) DNA and proteins.

**Additional file3: Fig. S1.** Electrophoresis of PCR products amplified from PC and BPH tissue specimens. PCR products were analyzed on a 2.5% agarose gel with molecular markers. Lane M: 50 bp DNA molecular ladder. Lane numbers represent the number of tissue samples from patients with prostate cancer (S1-PC panel a-d) and BHP (S1-BPH panel e, f). Lane P: The PCR products amplified from the JCPyV CY and BKPyV genomic DNA were used as positive controls and are indicated by arrows at 243 bp and 289 bp, respectively. PCR without a DNA template was used as a negative control. The PCR product of β-actin was used as an internal control (S1-PC panel a’-d’) and BHP tissues (S1-BPH panel e’, f’).

**Additional file 4: Fig. S2.** Schematic representation of the JCPyV and BKPyV regulatory regions identified in prostate cancer (a-d) and benign prostate hypertrophy (BPH) (e) tissues. Regulatory regions of JCPyV CY (a), JCPyV TW3 (b), JCPyV SK3 (c), JCPyV TW3 combined with BKPyV UT (d), and JCPyV CY combined with JCPyV TW3 (e) are shown for comparison. The numbers represent the number of tissue samples. (┥┝) deletion, (+)point mutation,(□)alteration.

## Data Availability

The datasets generated and/or analyzed during the current study are available in the NCBI GenBank repository, (a) https://www.ncbi.nlm.nih.gov/nuccore/9796397, accession No. AB03849; (b) https://www.ncbi.nlm.nih.gov/nuccore/M34049.1, accession No. M34049.1; (c) https://www.ncbi.nlm.nih.gov/nuccore/U61771, accession No. U61771; and (d) https://www.ncbi.nlm.nih.gov/nuccore/AB118652.1, accession No. AB118652.1, and their relevant details are mentioned in this published article.

## Abbreviations

PC: Prostate cancer
BPH: Benign prostate hypertrophy
JCPyV: Human polyomavirus John Cunningham virus
BKPyV: Human polyomavirus BK virus
TW3: JCPyV-TW3 strain
CY: JCPyV-CY strain
SK3: JCPyV-SK3 strain
UT: BKPyV-UT strain
LT: Large T antigen
IHC: Immunohistochemistry
TEM: Transmission electron microscopy
PSA: Prostate-specific antigen
CI: Confidence interval
OR: Odds ratio
AJCC: American Joint Committee on Cancer
TNM: Tumor-node-metastasis
