# Supplementary material for: The differential presence of human polyomaviruses, JCPyV and BKPyV, in prostate cancer and benign prostate hypertrophy tissues
Source: BMC Cancer. 2021 Oct 24;21:1141. doi: 10.1186/s12885-021-08862-w (PMC8543972; doi:10.1186/s12885-021-08862-w)
Supplement: Supplementary file 1 — Additional file 1: Table S1. Characteristics of prostate cancer (PC) samples and summary of the analysis of human polyomavirus (JCPyV and BKPyV) DNA and proteins. [file 12885_2021_8862_MOESM1_ESM.pdf]

Table S1. Characteristics of prostate cancer (PC) samples and summary of the analysis of human polyomavirus (JCPyV and BKPyV) DNA and proteins

Case No.	age (yrs)	Clinical features of PC			Detection methods			
		TNM	PSA (ng/mL)	Gleason score	PCR	sequencing	Immunohistochemistry (IHC) staining	
					DNA	genotype	LT	VP1
1	65	T3bN0	122	7(4+3)	-	ND	-	-
2	76	TxNxM1b	531.9	10(5+5)	+	TW3	+	+
3	68	T3bN0	7.8	8(4+4)	+	TW3	+	+
4	54	T3a	37.5	8(4+4)	-	ND	-	-
5	77	TxN1M1b	450	9(4+5)	-	ND	-	-
6	81	TxNxM0	>100	8(4+4)	-	ND	-	-
7	83	TxNxM1b	163	9(4+5)	-	ND	-	-
8	61	T3b	138	8(4+4)	+	TW3	+	+
9	63	TxN0M1c	3450	8(4+4)	+	TW3	-	+
10	74	T3a	41.4	9(4+5)	+	TW3	+	+
11	66	T2c	27.6	6(3+3)	-	ND	+	+
12	73	T2c	22.7	6(3+3)	+	TW3	+	+
13	93	T2cNxM1c	ND	9(4+5)	-	ND	-	-
14	83	TxNxM0	ND	7(3+4)	-	ND	-	-
15	84	T4	8.74	9(5+4)	-	ND	-	-
16	85	T3bN0	31.4	8(5+3)	-	ND	-	-
17	75	T3a	55.0	8(3+5)	+	TW3	+	+
18	81	T3b	375.0	9(4+5)	-	ND	-	-
19	71	T3bN0	25.3	8(4+4)	+	TW3	+	+
20	56	T3bN0	0.87	7(3+4)	-	ND	-	-
21	84	T4N0M1	209.5	9(5+4)	-	ND	-	-
22	70	T1b	34.7	8(4+4)	+	TW3	+	+
23	65	T2c	9.2	7(3+4)	-	ND	-	-
24	91	T2c	274.0	9(5+4)	-	ND	-	-
25	67	T3bN1	103.5	8(4+4)	-	ND	-	-
26	84	T3aN0	306.1	9(5+4)	-	ND	-	-
27	67	T4	143.1	9(5+4)	+	TW3	+	+
28	69	T3aN0	39.9	9(4+5)	+	CY	-	-
29	68	T3aN0	42.4	7(4+3)	-	ND	-	-
30	65	T3bN1	107.0	9(5+4)	+	TW3	+	+

31	62	T3bN1M1	45.5	8(4+4)	-	ND	-	-
32	61	T2cN0	13.8	9(4+5)	-	ND	-	-
33	67	T3aN1M1b	784.8	9(5+4)	-	ND	-	-
34	84	T3bN1	38.0	8(5+3)	-	ND	-	-
35	72	T3bN0	166.3	7(3+4)	-	ND	-	-
36	77	T3aN1M1b	177.79	8(4+4)	-	ND	-	-
37	74	T3aN0	78.8	8(4+4)	-	ND	-	-
38	73	T3bN0M0	52.7	9(4+5)	-	ND	-	-
39	82	T3bN0M0	690.1	7(4+3)	-	ND	-	-
40	65	T2cN0M0	11.9	7(4+3)	-	ND	-	-
41	68	TxNxM1	2050.0	7(3+4)	-	ND	-	-
42	71	T2cN0	11.8	7(4+3)	+	TW3	+	+
43	72	T3bN1M0	158.0	8(4+4)	-	ND	-	-
44	61	T2cN0M0	193.1	7(3+4)	-	ND	-	-
45	66	T3aN0M0	15.9	7(3+4)	-	ND	-	-
46	75	T3b,N0,M0	22.6	7(3+4)	+	TW3	+	+
47	78	T3bN1M1b	11200.0	9(4+5)	+	TW3	+	+
48	80	T2cN1M1b	1000.0	8(4+4)	+	TW3	+	+
49	78	TxNxM1b	404.5	8(4+4)	-	ND	-	-
50	81	T1N0M0	13.8	8(4+4)	+	TW3	+	+
51	69	T2cN0M0	18.7	7(3+4)	+	TW3	+	+
52	66	T3bN0M0	51.2	7(3+4)	+	TW3	+	+
53	75	T3bN1M0	767.0	7(3+4)	-	ND	+	+
54	79	T3bN1M0	1000.0	9(4+5)	+	TW3	+	+
55	79	T3bN0M1b	61.2	7(3+4)	-	ND	-	-
56	60	T3aN0	24.0	9(4+5)	+	TW3 & UT	+	+
57	66	T3b N1 M1b	183.0	8(3+5)	+	TW3&UT	+	+
58	85	TxNxM1b	13600.0	8(4+4)	-	ND	-	-
59	93	T3bN1M1c	>1000	7(3+4)	-	ND	-	-
60	59	T3aN0	23.0	7(4+3)	-	ND	-	-
61	79	T3aN0	24.4	8(3+5)	-	ND	-	-
62	76	T3bN0	184.0	7(3+4)	-	ND	-	-
63	79	T3aN0M1b	30.0	8(4+4)	-	ND	-	-
64	91	TxNxM1b	76.9	8(4+4)	-	ND	-	-
65	67	T3bN0M0	70.7	9(4+5)	-	ND	-	-
66	66	T4	38.9	7(3+4)	-	ND	-	-
67	82	T4	110.0	9(4+5)	+	TW3	+	+
68	80	T3bN0	82.3	7(4+3)	+	SK3	+	+

69	60	T2N0M0	5.9	8(4+4)	-	ND	-	-
70	77	TxNxM1b	213.9	10(5+5)	-	ND	-	-
71	81	TxN1M0	347.0	10(5+5)	-	ND	-	-
72	93	T4N1M1a	23.5	9(5+4)	-	ND	-	-
73	70	T3bN1M1b	68.8	7(4+3)	+	TW3	+	+
74	71	T4N1M1b	322.0	7(4+3)	-	ND	-	-
75	64	T3aN0M1b	23.0	7(4+3)	+	TW3 & UT	+	+
76	66	T3aN0	11.7	7(4+3)	+	TW3	+	+

"+": positive. "-": negative. ND: not detected. CY: JCPyV-CY strain (GenBank accession No.AB03849). TW3: JCPyV-TW3 strain-like (GenBank accession No.U61771).

SK3: JCPyV-SK3 strain-like (GenBank accession No. AB118256.1). UT: BKPyV-UT strain (GenBank accession No.M34049.1).

TNM: prostate cancer staging was determined by the 2018 AJCC TNM classification of malignant tumors. Tx: Tumor cannot be assessed. T1: The doctor cannot feel the tumor or see it with an imaging test. T1b: tumor was > 5% of the prostate. T2: Tumor has not yet spread outside of the prostate. T2c: Tumor present in both sides of the prostate. T3: Tumor has grown outside of the prostate and might have spread to the seminal vesicles. T3a: Extraprostatic extension. T3b: Seminal vesicle invasion. T4: Tumor has spread into other tissues next to the prostate. Nx: Lymph nodes in the region could not be assessed. N0: Tumor has not spread to nearby lymph nodes. N1: Tumor has spread to nearby lymph nodes. M0: Tumor elsewhere in the body. M1b: Tumor has spread to bones. (https://www.cancer.org/cancer/prostate-cancer/detection-diagnosis-staging/staging.html).

Tumor grade was detected by the Gleason score and Gleason grade. PSA: prostate specific antigen.
