# Supplementary material for: The differential presence of human polyomaviruses, JCPyV and BKPyV, in prostate cancer and benign prostate hypertrophy tissues
Source: BMC Cancer. 2021 Oct 24;21:1141. doi: 10.1186/s12885-021-08862-w (PMC8543972; doi:10.1186/s12885-021-08862-w)
Supplement: Supplementary file 2 — Additional file 2: Table S2. Characteristics of benign prostate hypertrophy (BPH) samples and summary of the analysis of human polyomavirus (JCPyV and BKPyV) DNA and proteins. [file 12885_2021_8862_MOESM2_ESM.pdf]

Table S2. Characteristics of benign prostate hypertrophy (BPH) samples and summary of the analysis of human polyomavirus (JCPyV and BKPyV) DNA and proteins

Case No.	Age (yrs)	Features of BPH	Detection methods			
			PCR	sequencing	Immunohistochemistry (IHC) staining	
		PSA (ng/mL)	DNA	genotypes	LT	VP1
1	73	1.3	-	ND	-	-
2	78	7.8	-	ND	-	-
3	68	2.7	-	ND	-	-
4	80	8.8	-	ND	-	-
5	76	6.9	-	ND	-	-
6	80	3.4	-	ND	-	-
7	74	14.8	-	ND	-	-
8	62	4.9	+	CY	-	-
9	71	5.6	-	ND	-	-
10	86	7.2	-	ND	-	-
11	69	5.4	-	ND	-	-
12	59	5.0	-	ND	-	-
13	71	16.3	-	ND	-	-
14	73	3.6	-	ND	-	-
15	74	5.8	-	ND	-	-
16	75	6.0	-	ND	-	-
17	72	0.1	-	ND	-	-
18	60	10.7	-	ND	-	-
19	74	8.8	+	ND	-	-
20	69	4.7	-	ND	-	-
21	73	5.9	-	ND	-	-
22	63	3.6	-	ND	-	-
23	68	7.6	-	ND	-	-
24	76	9.3	-	ND	-	-
25	69	7.2	-	ND	-	-
26	66	1.11	-	ND	-	-
27	70	4.6	-	ND	-	-
28	70	8.4	-	ND	-	-
29	67	8.2	+	TW3	-	-

30	69	21.5	-	ND	-	-

"+": positive. "-": negative. ND: not detected. CY: JCPyV-CY strain (GenBank accession No.AB03849). TW3: JCPyV-TW3 strain-like (GenBank accession No.U61771). PSA: prostate specific antigen.
