# Supplementary material for: The differential presence of human polyomaviruses, JCPyV and BKPyV, in prostate cancer and benign prostate hypertrophy tissues
Source: BMC Cancer. 2021 Oct 24;21:1141. doi: 10.1186/s12885-021-08862-w (PMC8543972; doi:10.1186/s12885-021-08862-w)
Supplement: Supplementary file 3 — Additional file3: Fig. S1. Electrophoresis of PCR products amplified from PC and BPH tissue specimens. PCR products were analyzed on a 2.5% agarose gel with molecular markers. Lane M: 50 bp DNA molecular ladder. Lane numbers represent the number of tissue samples from patients with prostate cancer (S1-PC panel a-d) and BHP (S1-BPH panel e, f). Lane P: The PCR products amplified from the JCPyV CY and BKPyV genomic DNA were used as positive controls and are indicated by arrows at 243 bp and 289 bp, respectively. PCR without a DNA template was used as a negative control. The PCR product of β-actin was used as an internal control (S1-PC panel a’-d’) and BHP tissues (S1-BPH panel e’, f’). [file 12885_2021_8862_MOESM3_ESM.pdf]

S1-PC

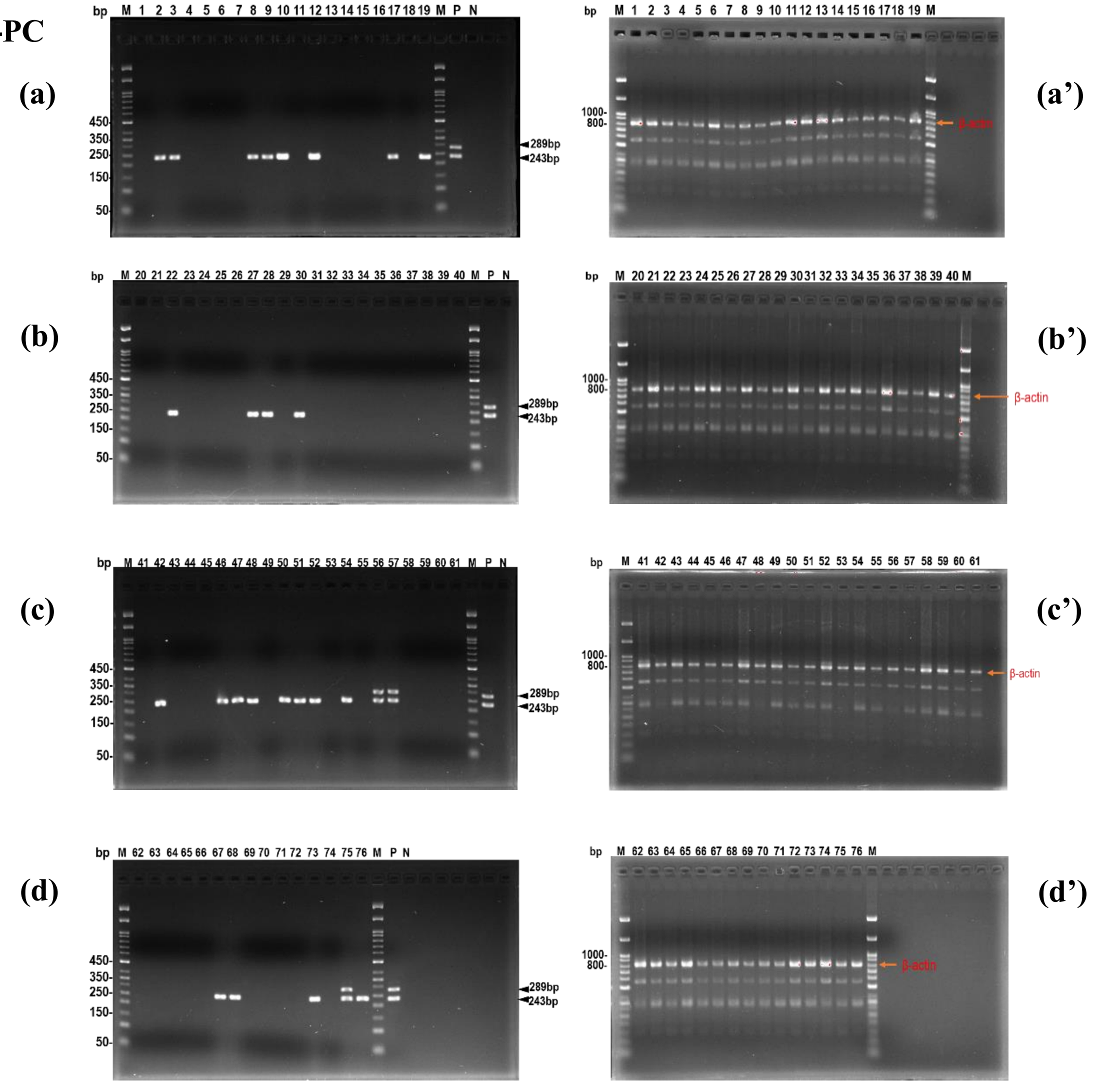

(a)
(a')
(b)
(b')
(c)
(c')
(d)
(d')
bp
M
P
N
450-
350-
250-
150-
50-
289bp
243bp
1000-
800-
β-actin

S1-BPH

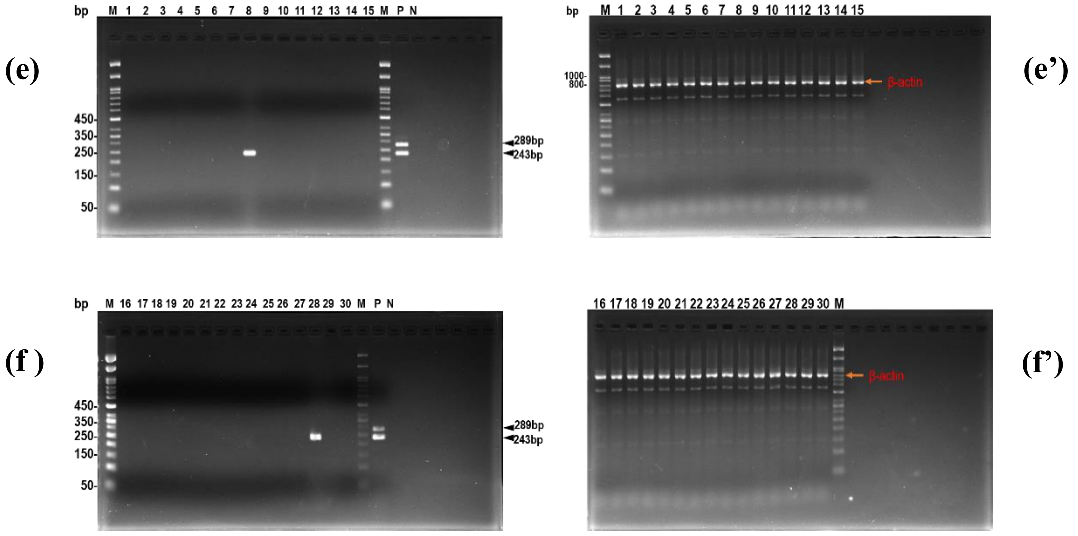

(e)
(e')
(f)
(f')
bp
M
P
N
450-
350-
250-
150-
50-
289bp
243bp
1000-
800-
β-actin

Fig S1. Detection of JCPyV and BKPyV viral DNA in prostate cancer and benign prostate hypertrophy specimens (original gel figures). The viral regulatory region was detected by nested PCR using the conserved JCPyV and BKPyV primers. The PCR products were analyzed on a 2.5% agarose gel with molecular markers. Lane M: 50 bp DNA molecular ladder. Lane numbers represent the number of the tissue samples from prostate cancer (S1-PC panel a-d) and BHP tissues (S1-BPH panel e,f). Lane P: The PCR products amplified from the JCPyV CY and BKPyV genomic DNA were used as positive controls and are indicated by arrows at 243 bp and 289 bp, respectively. PCR without a DNA template was used as a negative control. The PCR product of β-actin was used as an internal control (S1-PC panel a'-d') and BHP tissues (S1-BPH panel e', f').
