# Supplementary material for: The differential presence of human polyomaviruses, JCPyV and BKPyV, in prostate cancer and benign prostate hypertrophy tissues
Source: BMC Cancer. 2021 Oct 24;21:1141. doi: 10.1186/s12885-021-08862-w (PMC8543972; doi:10.1186/s12885-021-08862-w)
Supplement: Supplementary file 4 — Additional file 4: Fig. S2. Schematic representation of the JCPyV and BKPyV regulatory regions identified in prostate cancer (a-d) and benign prostate hypertrophy (BPH) (e) tissues. Regulatory regions of JCPyV CY (a), JCPyV TW3 (b), JCPyV SK3 (c), JCPyV TW3 combined with BKPyV UT (d), and JCPyV CY combined with JCPyV TW3 (e) are shown for comparison. The numbers represent the number of tissue samples. (┥┝) deletion, (+)point mutation,(□)alteration. [file 12885_2021_8862_MOESM4_ESM.pdf]

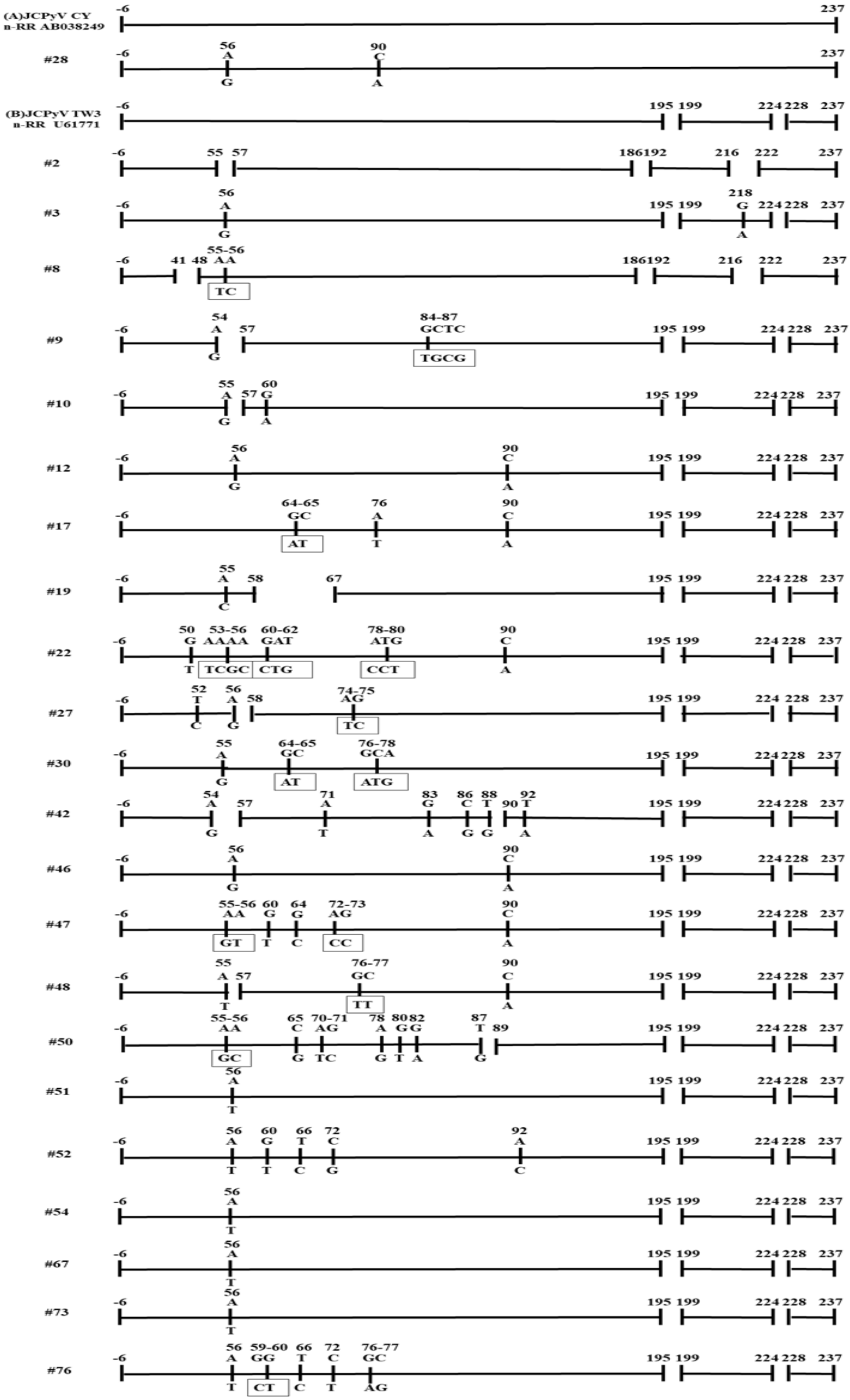
(A)JCPyV CY
n-RR AB038249
-6 237
#28
-6 56 A G 90 C A 237
(B)JCPyV TW3
n-RR U61771
-6 195 199 224 228 237
#2
-6 55 57 186 192 216 222 237
#3
-6 56 A G 195 199 218 G A 224 228 237
#8
-6 41 48 55-56 AA TC 186 192 216 222 237
#9
-6 54 A G 57 84-87 GCTC TGCG 195 199 224 228 237
#10
-6 55 A G 57 60 G A 195 199 224 228 237
#12
-6 56 A G 90 C A 195 199 224 228 237
#17
-6 64-65 GC AT 76 A T 90 C A 195 199 224 228 237
#19
-6 55 A C 58 67 195 199 224 228 237
#22
-6 50 G T 53-56 AAAA TCGC 60-62 GAT CTG 78-80 ATG CCT 90 C A 195 199 224 228 237
#27
-6 52 T C 56 A G 58 74-75 AG TC 195 199 224 228 237
#30
-6 55 A G 64-65 GC AT 76-78 GCA ATG 195 199 224 228 237
#42
-6 54 A G 57 71 A T 83 G A 86 C G 88 T G 90 92 T A 195 199 224 228 237
#46
-6 56 A G 90 C A 195 199 224 228 237
#47
-6 55-56 AA GT 60 G T 64 G C 72-73 AG CC 90 C A 195 199 224 228 237
#48
-6 55 A T 57 76-77 GC TT 90 C A 195 199 224 228 237
#50
-6 55-56 AA GC 65 C G 70-71 AG TC 78 A G 80 G T 82 G A 87 T G 89 195 199 224 228 237
#51
-6 56 A T 195 199 224 228 237
#52
-6 56 A T 60 G T 66 T C 72 C G 92 A C 195 199 224 228 237
#54
-6 56 A T 195 199 224 228 237
#67
-6 56 A T 195 199 224 228 237
#73
-6 56 A T 195 199 224 228 237
#76
-6 56 A T 59-60 GG CT 66 T C 72 C T 76-77 GC AG 195 199 224 228 237

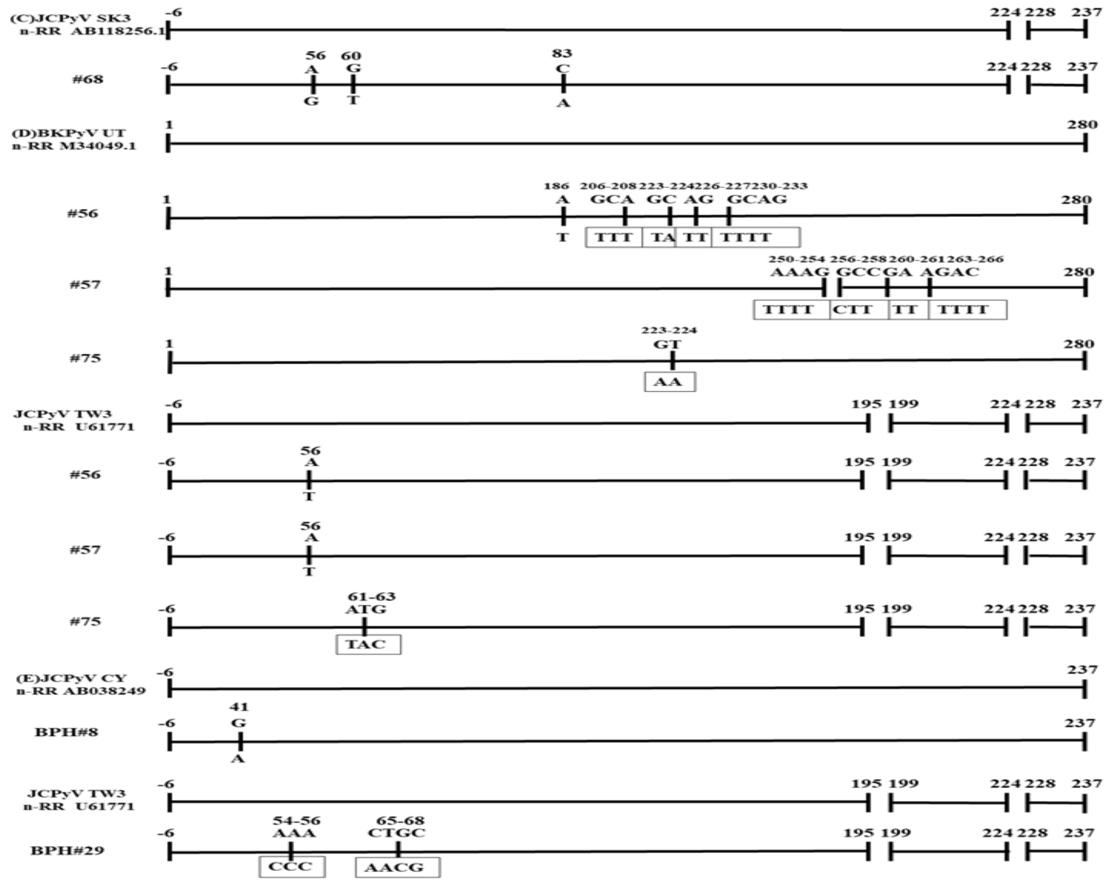

Fig. S2 Schematic representation of the JCPyV and BKPyV regulatory region identified in prostate cancer (a-d) and benign prostate hypertrophy (BPH) (e) tissues. Regulatory regions of JCPyV CY (a), JCPyV TW3 (b), JCPyV SK3 (c), JCPyV TW3 combined with BKPyV UT (d), and JCPyV CY combined with JCPyV TW3 (e) are shown for comparison. The numbers represent the number of tissue samples. (⊣ ⊢) deletion,(+)point mutation,(▭)alteration.
